# Bromodomain and Extraterminal Domain (BET) Protein Inhibition Hinders Glioblastoma Progression by Inducing Autophagy-Dependent Differentiation

**DOI:** 10.3390/ijms24087017

**Published:** 2023-04-10

**Authors:** Mayra Colardo, Deborah Gargano, Miriam Russo, Michele Petraroia, Daniele Pensabene, Giuseppina D’Alessandro, Antonio Santoro, Cristina Limatola, Marco Segatto, Sabrina Di Bartolomeo

**Affiliations:** 1 Department of Biosciences and Territory, University of Molise, 86090 Pesche, Italy; 2 Department of Science, University Roma Tre, 00146 Rome, Italy; 3 Department of Physiology and Pharmacology, Laboratory Affiliated to Istituto Pasteur Italia, Sapienza University of Rome, 00185 Rome, Italy; 4 Neuromed IRCCS, Via Atinense, 86077 Pozzilli, Italy; 5 Department of Human Neuroscience, Sapienza University of Rome, 00185 Rome, Italy

**Keywords:** BRD4, BRD2, differentiation, autophagy, GBM, epigenome

## Abstract

Glioblastoma multiforme (GBM) is the most common and aggressive type of malignant primary brain tumor, and it is characterized by a high recurrence incidence and poor prognosis due to the presence of a highly heterogeneous mass of stem cells with self-renewal capacity and stemness maintenance ability. In recent years, the epigenetic landscape of GBM has been explored and many epigenetic alterations have been investigated. Among the investigated epigenetic abnormalities, the bromodomain and extra-terminal domain (BET) chromatin readers have been found to be significantly overexpressed in GBM. In this work, we investigated the effects of BET protein inhibition on GBM cell reprogramming. We found that the pan-BET pharmacological inhibitor JQ1 was able to promote a differentiation program in GBM cells, thus impairing cell proliferation and enhancing the toxicity of the drug Temozolomide (TMZ). Notably, the pro-differentiation capability of JQ1 was prevented in autophagy-defective models, suggesting that autophagy activation is necessary for BET protein activity in regulating glioma cell fate. Given the growing interest in epigenetic therapy, our results further support the possibility of introducing a BET-based approach in GBM clinical management.

## 1. Introduction

GBM is the most common and aggressive type of malignant primary brain tumor, and it is characterized by a high recurrence incidence and poor prognosis [1,2]. Despite intense research efforts, the overall survival of patients GBM has not changed significantly in the last thirty years [3,4]. Indeed, GBM tends to recur despite multi-modal therapies, with a relapse rate as high as 90% and with less than 10% of patients surviving at 5 years post-diagnosis [5,6]. According to the common view, the driving force sustaining its tumor growth, resistance to treatment, and recurrence is likely the presence of a heterogeneous tumoral mass of a small population of stem cells (GCS) characterized by self-renewal capacity and stemness maintenance ability [7,8,9]. 

For this reason, the elucidation of the molecular mechanisms controlling GSCs has aroused great interest in obtaining novel therapeutical strategies.

Unlike other brain cancers, GBM contains neural precursors endowed with features expected from neural stem cells (NSCs) [10]. There are significant similarities between NSCs and GSCs, such as the expression of stem cell markers and the ability to differentiate into neuron- and glial-like cells, showing aberrant and mixed neuronal/astroglial phenotypes in cultures [10,11]. However, GSCs harbor genetic abnormalities that contribute to tumor invasion, angiogenesis, and radio-resistance [12]. In recent years, the epigenetic landscape of GBM has been also explored and many epigenetic alterations, such as histone modification, DNA methylation, and chromatin remodeling have been investigated [7,13]. Among the epigenetic abnormalities, bromodomain and extraterminal domain (BET) chromatin readers have been found to be significantly overexpressed in GBM tissue compared to normal brain tissue [14,15,16]. Furthermore, it has been observed that BET bromodomain inhibitors can inhibit the transcription of c-Myc. Subsequently, they have attracted increasing interest as valuable candidates for the clinical treatment of Myc-driven cancer, including GBM [17,18,19]. Recent studies have demonstrated that pharmacological BET inhibition is effective in counteracting GBM growth in both in vitro and in vivo models, similar to what has been observed in other tumor models [20,21,22,23]. However, the molecular mechanisms of BET proteins in GBM tumorigenesis are scarcely understood, and the potential of BET inhibitors in treating GBM is largely unexplored.

Intriguingly, the BET member BRD4 has been found to act as a transcriptional repressor of the autophagic process [24]. In detail, BRD4 suppresses the expression of a subset of autophagy and lysosome genes by binding to promoter regions under normal growth conditions, and its inhibition enhances autophagic flux and lysosomal function [24]. 

The role of autophagy in cancer onset and progression remains controversial as it may promote or hinder tumor progression depending on the tumor type and stage. In GBMs, autophagy has been often associated with chemoresistance mechanisms, although growing evidence has also indicated that autophagy induction can counteract GBM proliferation and invasiveness [25,26,27,28,29].

It has been demonstrated that autophagy modulates the proliferation and differentiation of normal neuronal stem cells (NSCs), as well as NSC niche maintenance; on the contrary, its failure may contribute to GSC expansion and maintenance [30,31].

In this work, we investigated the impact of BET protein inhibition on GBM cell reprogramming in both in vitro and in ex vivo models. We found that the pan-BET pharmacological inhibitor JQ1 was able to promote a differentiation program in GBM cells, likely toward a neuronal-like phenotype, thus impairing cell proliferation and sensitizing cells to the drug Temozolomide (TMZ). Notably, the pro-differentiation capability of JQ1 was less evident in autophagy-defective models, suggesting that autophagy activation is necessary for BET protein activity in regulating glioma cell fate.

## 2. Results

### 2.1. BRD2 and BRD4 Are Expressed in GBM Cells 

**Figure 1:**
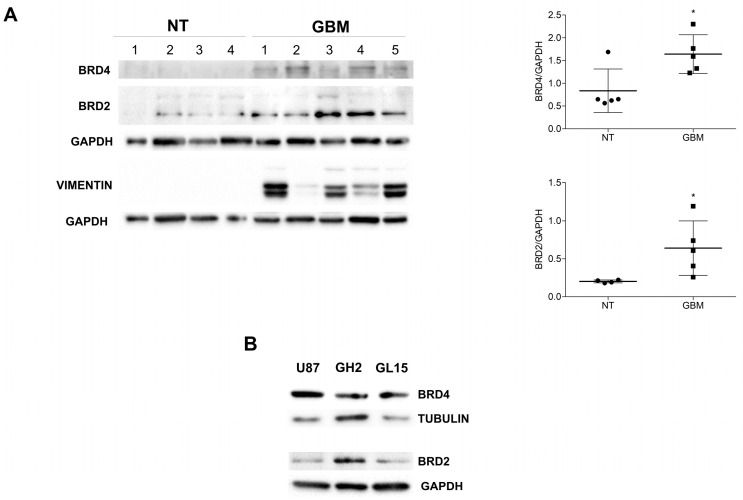
BRD protein expression in GBM tissues and cells. (A) Protein extracts from non-tumoral surgical cerebral tissues (NT) and from GBM specimens (GBM) were subjected to Western blotting analysis for BRD4 and BRD2 proteins by using specific antibodies. VIMENTIN expression was used as a tumorigenicity control and GAPDH as a loading control. Densitometric analyses of BRD4 and BRD2 expression are shown in the graphs. The sum of all the bands of the BRD2 protein was analyzed. Statistical significance: * *p* < 0.05, Student t-test and. (**B**) Western botting analysis of BRD4 and BRD2 was performed on protein extracts from U87, GH2, and GL15 cells, and a representative image is shown.

Abnormal BET mRNA and protein expression has been previously observed in glioma in vitro and ex vivo models. Western blotting analysis from GBM patient biopsies highlighted a significant increase in the expression levels of the BRD4 and BRD2 proteins when compared to non-tumoral ones (Figure 1A). We also confirmed BRD4 and BRD2 protein expression in our in vitro experimental models, namely, in the U87MG (U87, hereafter) and GL15 cell lines and in a patient-derived primary cell line (GH2) (Figure 1B). 

### 2.2. The BETi JQ1 Hampers Proliferation and Induces Apoptosis in Combination with Temozolomide in GBM Cells 

**Figure 2:**
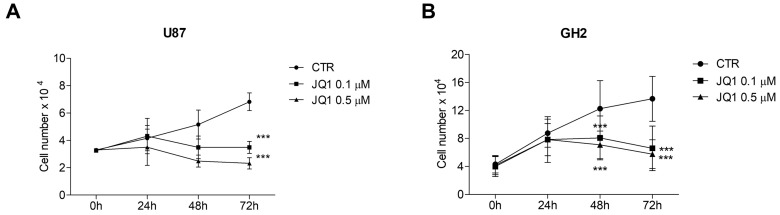
BET protein inhibition leads to cell proliferation arrest and G1 accumulation. U87 (**A**) and GH2 (**B**) cells were cultured in complete DMEM (0 h) or in the presence of 0.1 or 0.5 μM of JQ1. At the indicated time points, the cells were trypsinized and counted in a Thoma chamber. The graph represents the means ± SEMs of three different experiments. Statistical significance: *** *p* ≤ 0.001, two-way ANOVA.

It has been reported that BET protein inhibition by small synthetic molecules is able to inhibit GBM cell proliferation in in vitro and in vivo models. In order to investigate the effect of BET inhibition on GBM tumorigenesis, we took advantage of the pan-BET inhibitor JQ1. Firstly, to test the effect of JQ1 on cell proliferation and viability in our cellular models, we performed cell counts on U87 and GH2 cells grown in the presence of 0.1 and 0.5 μM of JQ1 for 24, 48, and 72 h. JQ1 significantly affected cell proliferation beginning at 48 h of treatment for both the concentrations employed and in both cell models (Figure 2A,B). 

**Figure 3:**
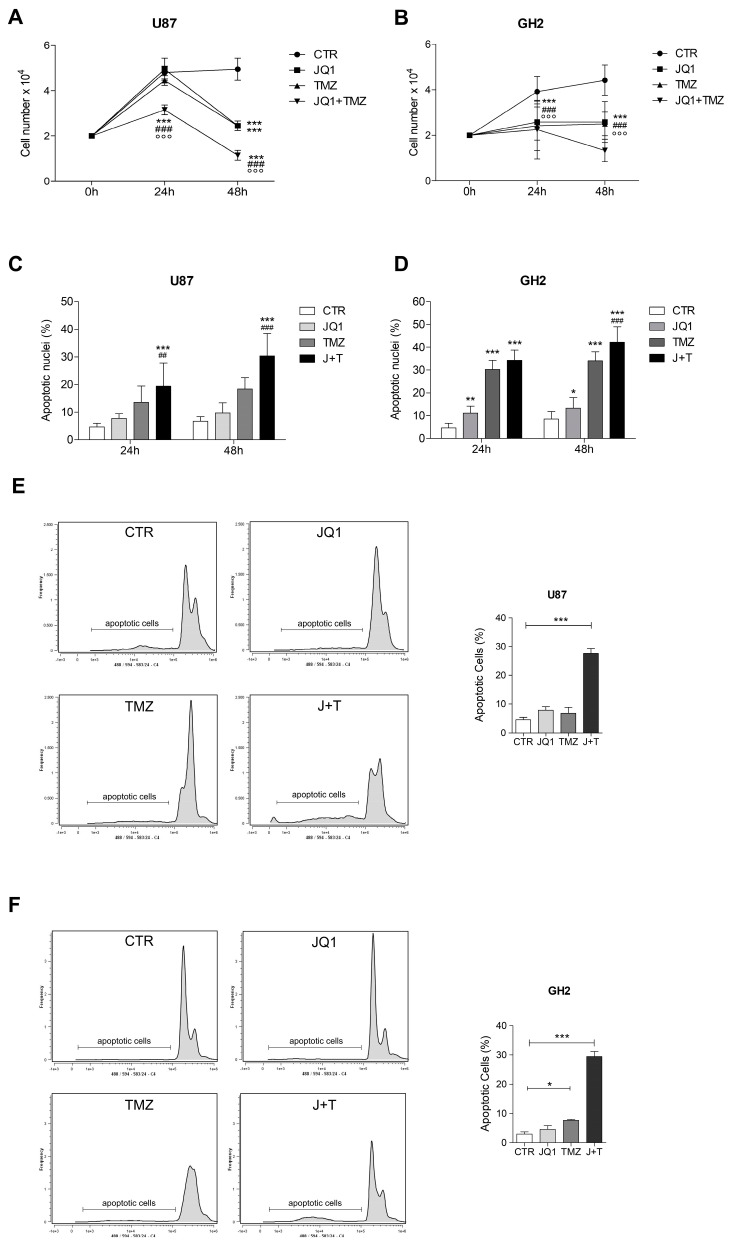
JQ1 sensitizes cells to TMZ. U87 (**A**) and GH2 (**B**) cells were grown in complete DMEM (CTR) or in the presence of 0.5 μM of JQ1, 500 μM of TMZ, or both, as indicated. At the indicated time points, the cells were trypsinized and counted in a Newbauer chamber. The graph represents the means ± SEMs of three different experiments. Statistical significance: * indicates significance vs. CTR; # indicates significance vs. TMZ; ° indicates significance vs. JQ1; ## *p* < 0.01, ### *p* ≤ 0.001, °°° *p* ≤ 0.001 and *** *p* ≤ 0.001, two-way ANOVA. The U87 (**C**) and GH2 (**D**) cells cultured as in (**A**) and (**B**) were fixed, stained with DAPI, and counted under a fluorescence microscope (using 20X objective). At least 10 fields per each condition were counted. The graphs represent the means ± SDs of three different experiments. * indicates significance vs. CTR; # indicates significance vs. TMZ; and * *p* < 0.05, ** *p* < 0.01, and *** *p* ≤ 0.001, two-way ANOVA. (**E**,**F**) The U87 (**E**) and GH2 cells (**F**) were cultured as in (**A**,**B**) for 48 h, and PI-FACS analysis was performed. A PI-positive, subG1 population representing the apoptotic cells is indicated in each panel and in the representative graphs. A total of 10000 cells were counted. The graphs represent the means ± SDs of three different experiments. * indicates significance vs. CTR, and * *p* < 0.05 and *** *p* ≤ 0.001, two-way ANOVA.

Then, we investigated the effect of JQ1 stimulation on cells treated with Temozolomide (TMZ), the first-line chemotherapeutic agent for GBM treatment. As shown in Figure 3, TMZ impaired cell proliferation beginning at 48 h of stimulation, and JQ1 co-administration significantly enhanced its anti-proliferative activity in both U87 and GH2 cells (Figure 3A and Figure 3B, respectively). Nuclear staining with DAPI and FACS analysis revealed that JQ1 itself induced a slight increase in apoptosis up to 48 h of stimulation, which was significant in GH2 cells only, and TMZ alone also induced a significant increase in apoptotic cells, as has been reported. However, the combination of JQ1 and TMZ strongly promoted apoptosis, especially beginning at 48 h of treatment in both cell lines (Figure 3C–F).

### 2.3. BET Inhibition Induces the Differentiation of GBM Cells

**Figure 4:**
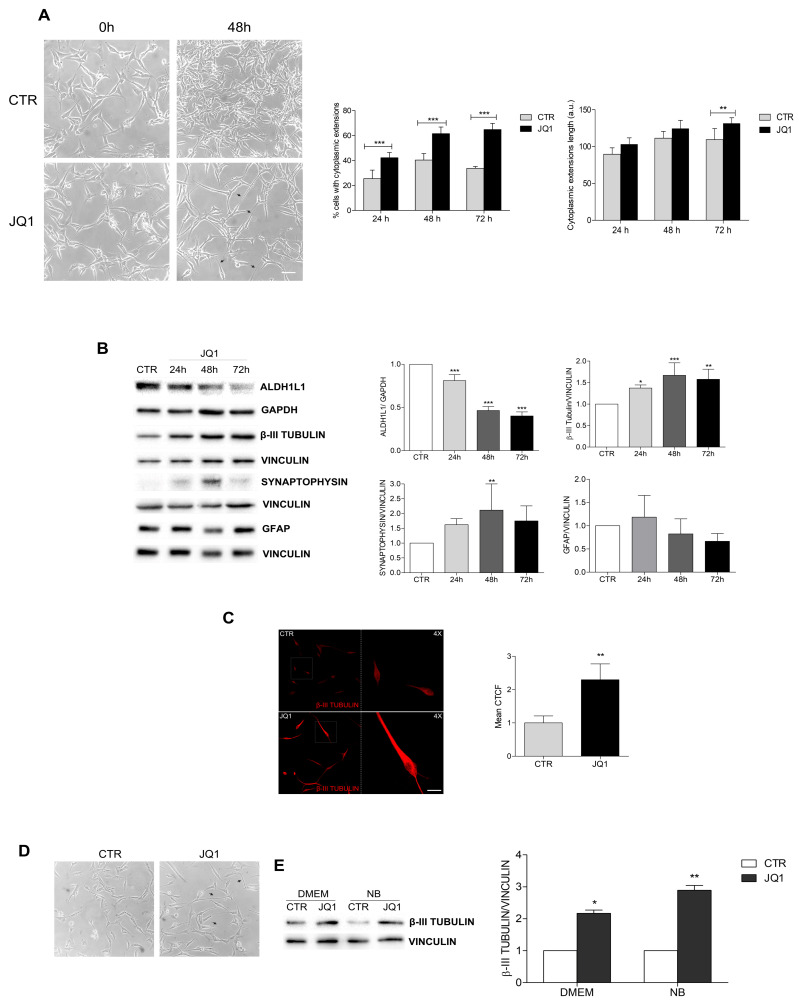
BET inhibition induces differentiation in U87 cells. (**A**) U87 cells were cultured in complete DMEM (CTR) or in the presence of 0.5 μM of JQ1 and phase contrast pictures at 20× magnification were taken at 24, 48, and 72 h. Representative images of the cells upon 48 h of JQ1 stimulation are shown. The arrow heads in the pictures indicate cytoplasmic extensions. Scale bar: 100 µm. The percentage of cells with cytoplasmic extensions and the extension length were calculated by Image J analysis and are shown in the graphs. At least 10 fields per each condition were counted. ** *p* < 0.01 and *** *p* ≤ 0.001, two-way ANOVA. (**B**) Western botting analysis was performed on the protein extracts of the U87 cells cultured as in (**A**). Specific antibodies for ALDH1, βIII-TUBULIN, SYNAPTOPHISIN, and GFAP were used, and representative images are shown. GAPDH, VINCULIN, and α-TUBULIN were used as loading controls. The corresponding densitometric analyses are shown in the graphs. Statistical significance: * *p* < 0.05, ** *p* < 0.01, *** and *p* ≤ 0.001, one-way ANOVA. (C) Immunocytochemistry and confocal analysis for β3-TUBULIN expression (red) was performed on the U87 cells after 48 h of treatment with 0.5 μM of JQ1. Scale bar: 30 μM. A 4× magnification is shown in the right panel. Analysis of the mean corrected total cell fluorescence (CTCF) was performed by Image J software on 10 fields per each condition and the corresponding graph is shown. ** *p* < 0.01, Student’s *t*-test. (**D**) U87 cells were cultured as a monolayer in Neurobasal/B27 medium for 8 h before stimulation with 0.5 μM of JQ1. After 48 h, phase contrast pictures at 20× magnification were taken. The arrow heads in the pictures indicate cytoplasmic extensions. Scale bar: 100 µm. (**E**) U87 cells grown in DMEM or in Neurobasal medium (NB) were treated with 0.5 μM JQ1 for 48 h. The corresponding protein extracts were subjected to western blotting analysis for βIII-TUBULIN and Vinculin was used as loading control. Densitometric analysis of the blot is shown in the graph. * *p* < 0.05, ** *p* < 0.01, one-way ANOVA.

Surprisingly, the JQ1-treated U87 cells showed marked morphological changes when compared to the untreated cells. In detail, an increase in cells bearing cytoplasmic extensions and an elongation of cytoplasmic extensions was observed, beginning at 24 h, and this was more evident at 48 and 72 h (Figure 4A). The same effect was observed in the GH2 cells (Supplementary Figure S1). The increases in the number and length of cytoplasmic extensions, together with the arrest of cell proliferation, suggested the induction of a differentiation process by JQ1. To test this hypothesis, we analyzed the expression levels of the stemness marker ALDH1L1 and observed its drastic reduction after 48 h of JQ1 stimulation in both the U87 and GH2 cells (Figure 4B and Supplementary Figure S1). On the contrary, an increased expression of β3-tubulin and of synaptophysin, two markers of neuronal cells, was observed in the JQ1-treated cells (Figure 4B and Supplementary Figure S1). Notably, the analysis of the expression of the glial marker GFAP did not show any difference among the control and JQ1-treated cells (Figure 4B and Supplementary Figure S1). β3-tubulin accumulation was further confirmed by immunofluorescence experiments in both cell lines (Figure 4C and Supplementary Figure S1). To rule out the possibility of serum interference on JQ1 effects, we also performed JQ1 treatment in the presence of Neurobasal medium. Elongation of the cytoplasmic extensions and β3-tubulin accumulation were also observed in this experimental setting, as shown in Figure 4D. Altogether, these results appear to suggest that BET inhibition could stimulate cell differentiation, likely toward a neuronal-like fate, in GBM cells. 

### 2.4. PI3K/Akt1/mTOR Pathway Is Inhibited and Autophagy Is Modulated by JQ1

**Figure 5:**
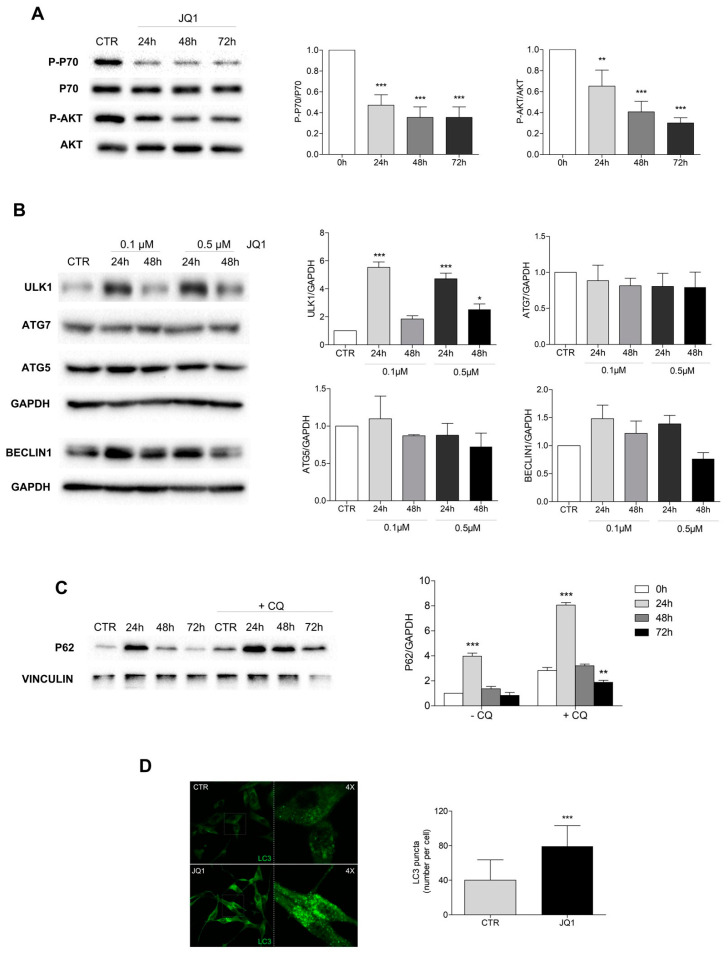
Autophagy is induced in JQ1-stimulated U87 cells. (**A**) U87 cells were cultured in complete DMEM (CTR) or in the presence of 0.5 μM of JQ1 for 24, 48, and 72 h, and the corresponding protein extracts were analyzed by Western blotting. The protein expression levels and activity of AKT1 and p70S6 were analyzed by using specific antibodies for the phosphorylated and total forms of both proteins. Representative images of three different experiments are shown. VINCULIN was used as a loading control. The corresponding densitometric analyses are shown in the graphs. Statistical significance: ** *p* < 0.01 and *** *p* ≤ 0.001, one-way ANOVA. (**B**) Western blotting analysis of the autophagy regulators ULK1, ATG7, ATG5, and BECLIN1 in the protein extracts of the U87 cells stimulated with 0.1 or 0.5 μM of JQ1 for 24 and 48 h. Representative images of three different experiments are shown. GAPDH was used as a loading control. The corresponding densitometric analyses are shown in the graphs. Statistical significance: *** *p* ≤ 0.001, one-way ANOVA. (**C**) Autophagy flux was analyzed in the cells stimulated as in (A) in the presence or absence of 20 μM of chloroquine (CQ). The expression of p62 is shown. VINCULIN was used as a loading control. The corresponding densitometric analyses are shown. Statistical significance: * *p* < 0.05 and ** *p* < 0.01, one-way ANOVA. (**D**) U87 cells treated with 0.5 μM of JQ1 for 48 h and untreated (CTR) cells were subjected to immunocytochemistry and confocal analysis for LC3 (green). Representative images of at least 10 fields showing the merged signals are shown. Scale bar: 30 μM. A 4× magnification is shown in the right panel. The LC3 puncta per cell were counted in 30 cells for each condition, and the corresponding graph is shown. Statistical significance: *** *p* ≤ 0.001, Student’s *t*-test.

It has been reported that JQ1 suppresses tumor growth through the regulation of the PI3K/AKT1/mTOR pathway in several cancer models, including GBM. We analyzed the activity of the AKT1 and mTOR kinases by means of specific phospho-antibodies, and we observed a significant dephosphorylation of AKT1 and the mTOR substrate p70S6K in the U87 cells treated with 0.5μM of JQ1, especially after 48 and 72 h of stimulation (Figure 5A). 

As Akt/mTOR also regulates autophagy, we also investigated the effect of JQ1 stimulation on the autophagy process in our models. Western blotting analyses indicated that JQ1 modulates the protein expression of some autophagy players in U87 and GH2 cells (Figure 5B and Supplementary Figure S2A). In detail, a transient up-regulation of the autophagy regulator Ulk1 was observed at both 0.1 and 0.5 μM of JQ1, whereas no significant differences were revealed in the protein levels of ATG5, ATG7, and BECLIN 1 (Figure 5B and Supplementary Figure S2A). A transient up-regulation of the autophagy substrate p62 at 24 h of JQ1 treatment was also observed, followed by a dramatic decrease which was not prevented by blocking the autophagy flux with the inhibitor chloroquine (CQ), thus suggesting an impairment in protein expression instead of a protein degradation (Figure 5C and Supplementary Figure S2B). In order to clarify whether or not JQ1 was able to induce autophagy in our models, we performed an immunofluorescence analysis of the endogenous LC3. As shown in Figure 5D, an accumulation of LC3 dots was observed at 48 h of JQ1 stimulation in both the U87 and GH2 cells (Figure 5D and Supplementary Figure S2C).

### 2.5. JQ1-Induced Cell Differentiation Is Dependent on Autophagy 

**Figure 6:**
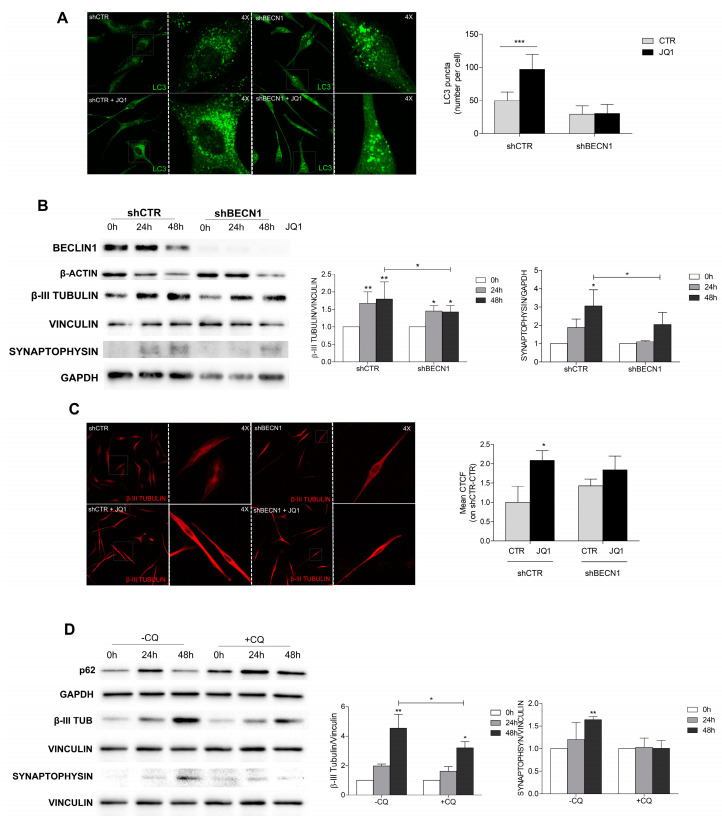
Autophagy-defective cells are less likely to differentiate than autophagy-proficient ones. (A) GL15 cells silenced for the BECN1 gene (shBECN1) or for a scramble gene (shCTR) were cultured in DMEM (CTR) or in the presence of 0.5 μM of JQ1 for 48 h and subjected to immunocytochemistry and confocal analysis for LC3 (green). Representative images of at least 10 fields showing the merged signals are shown. Scale bar: 30 μM. A 4× magnification is shown in the right panel. The LC3 puncta per cell were counted in 30 cells for each condition and the corresponding graph is shown. Statistical significance: ** *p* < 0.01 and *** *p* ≤ 0.001, Student’s *t*-test. (**B**) Western blot analysis of β3-TUBULIN and SYNAPTOPHISIN was performed in shBECN1 and shCTR GL15 cells cultured in the presence of 0.5 μM of JQ1 or in DMEM medium (CTR) for 24 and 48 h. A specific antibody for BECLIN1 was used to confirm the silencing efficiency. GAPDH, VINCULIN, and β-ACTIN were used as loading controls. Each blot is representative of three independent experiments. The corresponding densitometric analyses are shown in the graphs. Statistical significance: * *p* < 0.05, ** *p* < 0.01 and *** *p* ≤ 0.001, two-way ANOVA. (**C**) Immunocytochemistry and confocal analysis for β3-TUBULIN expression (red) was performed in shCTR and shBECN1 cells grown alone (CTR) or in the presence of 0.5 μM of JQ1. Scale bar: 30 μM. A 4× magnification is shown in the right panel. Analysis of the mean corrected total cell fluorescence (CTCF) was performed by Image J software on 10 fields per each condition, and the corresponding graph is shown. Statistical significance: *** *p* ≤ 0.001, Student *t*-test. (**D**) GH2 cells were stimulated with of 0.5 μM of JQ1 for 24 and 48 h, and 20 μM of chloroquine (CQ) was added in the last 16 h. Western blotting analysis was performed on the protein extracts and the expression of β3-TUBULIN and SYNAPTOPHISIN was analyzed. The analysis of p62 was used to confirm the CQ efficiency in the autophagy impairment. Statistical significance: * *p* < 0.05, ** *p* < 0.01, two-way ANOVA.

It has been described that functional autophagy is required to promote differentiation and reduce the stemness properties of cancer stem cells [31,32]. By taking advantage of BECN1-devoid GBM cells [28,33], we investigated the capability of JQ1 to stimulate cell differentiation in an autophagy-defective setting. First, we verified the inability of JQ1 to induce autophagosome accumulation in sh-BECN1 GL15 cells and found no difference in the number of LC3 dots between the control and treated cells, unlike the effects observed on sh-CTR cells (Figure 6A). 

Notably, using Western blotting analysis, we found that the increase in β3-tubulin and synaptophysin, observed upon JQ1 treatment in the sh-CTR cells, was significantly less evident in the sh-BECN cells (Figure 6B). Immunostaining experiments also confirmed a slight increase in the β3-tubulin signal in the JQ1-treated sh-BECN cells in comparison to that observed in the sh-CTR cells (Figure 6C).

To further investigate the impact of autophagy on JQ1-induced cell differentiation, experiments were also performed on GH2 cells in the presence of the autophagy pharmacological inhibitor CQ, which is known to impair autophagosome–lysosome fusion in the last step of autophagy. According to the results obtained for the BECLIN 1-devoid cells, the β3-tubulin up-regulation induced by JQ1 treatment was hampered by CQ co-administration, as was synaptophysin expression (Figure 6D). Taken together, these experiments suggested that proper autophagy activation and completion are required for cell [34] reprogramming induced by BET protein inhibition. 

## 3. Discussion

The BET protein family consists of three members commonly expressed in mammals, which contain two tandem bromine domains that recognize and bind to acetylated lysine residues on histones H3 and H4 and on non-histone proteins [35,36]. Through binding to acetylated chromatin, BET proteins regulate transcription by the recruitment of several protein complexes on nucleosomes [37].

Among the four members, BRD4 has been found to be overexpressed in GBM cells, and it was inversely correlated to overall survival in patients in the TCGA and CGGA databases [14,38]. In this paper, we confirmed that the BRD4 protein was overexpressed in GBM specimens in comparison to non-tumoral cerebral tissue, and it was also expressed in both immortalized and primary GBM cells. In addition to BRD4, we found, for the first time, an increased expression of BRD2 protein in tumoral tissues compared to normal ones, according to the databank data showing the up-regulation of the specific transcript [14,38]. Conversely, we did not find BRD3 expression or modulation in either of the tissue and cell samples. The BRD2 and BRD4 up-regulation in the GBM models prompted us to better characterize the BET protein involvement in tumor progression, taking advantage of the well-characterized and specific pan-inhibitor JQ1 [36]. In accordance with other literature data, we observed that BET inhibition by JQ1 induced an arrest in cell proliferation and promoted apoptosis when co-administered with the drug TMZ. The drastic morphological change we observed prompted us to investigate the occurrence of a differentiation process in the JQ1-treated cells. Indeed, it has been previously demonstrated that BET members regulate the cell differentiation of stem cells through the involvement of several signal transduction pathways [2,39]. Moreover, BRD4 has been found to be concentrated at the Notch1 promoter region, thus modulating the Notch1 signaling pathway that is involved in the regulation of the self-renewal and tumorigenicity of glioma stem cells [16]. Notably, we observed that BET inhibition induced cell differentiation in the GBM in vitro models we analyzed, which was in line with the effect of BET inhibition previously observed on neural progenitor cells [40]. In detail, an increased expression of the neuronal markers β3-tubulin and synaptophysin, but not of the glial protein GFAP, was observed in the JQ1-treated cells. Moreover, a decrease in the protein ALDH1L1, which has been described as a marker of stemness in glioma models [41], was found. We also found the inactivation of the PI3K/Akt1/mTOR pathway in cells upon JQ1 stimulation, and as a consequence, we decided to explore the effect of BET inhibition on the autophagy process. Indeed, it has been recently demonstrated that BRD4 represses the transcriptional program that promotes autophagy [24]. By analyzing the expression of some autophagy-related proteins, we observed that JQ1 induces a marked and transient upregulation of the autophagy upstream regulator ULK1, whereas no significant modulation of the autophagy players ATG5, ATG7, and Beclin 1 was noted. A transient increase, followed by a strong decrease, in the autophagy substrate p62 was also observed. LC3-decorated autophagosomes were clearly observed under microscopy analysis in cells incubated with JQ1, thus indicating the autophagy activation occurrence, as has also reported in another cell model [42].

Autophagy plays a crucial role during embryonic development and differentiation, ensuring proper cell homeostasis and maintaining the stemness properties of self-renewal cells [43,44,45]. Since autophagy has been found to contribute to GSC expansion and maintenance, we analyzed the pro-differentiative activity of JQ1 in two autophagy-defective GBM models: a genetic model represented by a GBM cell line (GL15) silenced for the autophagy upstream regulator BECLIN1 [33] and a pharmacological model using the lysosomotropic agent CQ [46]. In both autophagy-defective models, a weaker buildup of neuronal markers was observed in comparison with the autophagy-proficient counterparts. This result indicated that autophagy activation is crucial for the activation of a pro-differentiative program toward a neuronal-like fate by JQ1. We may speculate that an abnormal BET overexpression negatively regulates autophagy in GBM cells, thus contributing to stemness maintenance and tumor aggressiveness. A direct reprogramming of GBM cell into a differentiated, non-proliferating fate could potentially represent an effective strategy, in combination with conventional therapy, for this very aggressive type of tumor.

Given the growing interest in epigenetic therapy, which consists of manipulating the deregulated epigenome of cancer cells, our results further support the possibility of introducing a BET-based approach in GBM clinical care.

## 4. Materials and Methods

### 4.1. Cell Culture and Treatments

Human GBM U87MG (U87) and GH2 cell lines were kindly provided by Prof. G. Velasco (Complutense University, Madrid, Spain). The GH2 were cells derived from a GBM patient and were obtained from the Spanish National Cancer Center (CNIO, Madrid, Spain) biobank (GH2). All procedures involving samples of human origin were performed with the approval of the corresponding ethical committees from each institution, as well as that of the ethical committee of Complutense University (Madrid, Spain). Histopathological typing was completed according to the WHO criteria and resulted as grade IV. Briefly, GIC cultures were obtained by using the following procedure: tumour samples were mechanically and enzymatically dissociated with 0.12 mg/mL of the collagenase type Ia from Clostridium histolyticum (#C9722, Sigma-Aldrich, St. Louis, MI, USA) for 2 h at 37 °C and filtered using a 100 μm nylon filter (Millipore, Burlington, MA, USA). Human GBM GL15 cells were kindly provided by Dr. E. Castigli of the University of Perugia, Italy. The GL15 shBECLIN1 and GL15 shCTR cells were prepared by lentiviral infection as previously described [33]. Human biotic specimens were obtained after patient surgeries at Policlinico Umberto I (Rome) from patients who provided written informed consent to the research proposals. The study was approved by the Institutional Ethics Committee of Sapienza University. Histopathological typing and tumor grading were completed according to the WHO criteria and resulted as grade IV. 

Human U87, GL15, and GH2 cells were cultured in DMEM (Lonza, Basel, Switzerland) and supplemented with 10% heat-inactivated FBS (Euroclone, Milan, Italy) and a 1% penicillin/streptomycin solution (Euroclone, Milan, Italy). The cells were grown at 37 °C in a 5% CO_2_ humidified atmosphere. Where indicated, the U87 and GH2 cells were cultured in Neurobasal medium in the presence of a B27 supplement. Neurobasal medium was added directly to monolayers or, alternatively, to cell suspensions to allow tumorsphere formation. We added 0.1 µM and 0.5 µM of JQ1 (Merck KGaA, Darmstadt, Germany) to the culture medium for 24, 28, and 72 h, as indicated. Cells treated with the vehicle (DMSO in cell culture media) served as the control. In order to inhibit autophagy and to monitor autophagy flux, 20 µM of CQ was added to the culture medium for the last 16 h of the JQ1 treatment. For the proliferation and apoptosis assays, 500 μM of Temozolomide (Merck KGaA, Darmstadt, Germany) was employed for the time indicated.

### 4.2. Cell Lysis and Western Blotting

The protein extracts were prepared by lysing cells with the appropriate amount of RIPA buffer (50 Mm Tris HCl, pH 7.4; Triton 1%; Na Deoxycholate 0.25%; SDS 0.1%; 150 mM NaCl; 1 mM EDTA; and 5 mM MgCl 2 supplemented with a protease inhibitor cocktail). After incubation on ice for 20 min, the samples were centrifuged at 160,000× *g* for 15 min at 4 °C. The supernatants were recovered and the protein concentrations were determined using a Lowry protein assay (Bio-Rad Laboratories, Milan, Italy). Laemmli buffer 5X (Tris-HCl 315 mM, pH 6.8; 2.5% β-mercaptoethanol; 50% glycerol; 10% sodium dodecyl sulfate; and 0.5% Bromophenol Blue) was added to supernatants and the samples were boiled at 95 °C for 5 min. The proteins extracts were separated on SDS-PAGE and then electroblotted onto nitrocellulose (GE Healthcare, Life Sciences, Little Chalfont, Buckinghamshire, UK) using a turbo trans-blot transfer system (Biorad Laboratories, Milan, Italy). After blocking with 5% fat-free milk powder in Tris-buffered saline and 0.1% Tween-20, the membranes were probed overnight at 4 °C with primary antibodies. Detection was obtained by using horseradish peroxidase-conjugated secondary antibody (Bio-Rad Laboratories, Milan, Italy), the protein antibody immunocomplexes were visualized with ECL plus (GE Healthcare, Life Sciences, Little Chalfont, Buckinghamshire, UK), and chemiluminescence was recorded using a ChemiDoc MP system (Bio-Rad Laboratories, Milan, Italy). The following primary antibodies were used: anti-BRD4, anti-BRD2, anti-ALDH1L1, anti-Synaptophysin, anti-β-III-Tubulin, anti-p62, anti-ULK1, anti-BECLIN1, anti-GAPDH, and anti-HSP90 (Santa Cruz Biotechnology, Santa Cruz, CA, USA), as well as anti-Akt, anti-p-Akt, anti- P-p70S6K, anti-p70S6K, anti-LC3B, anti-P-ERK1/2 (Thr 202/Tyr 204), anti-ERK1/2, (Cell Signaling, Danvers, MA, USA), anti-vinculin, and anti-β-Actin (Sigma Aldrich, Milan, Italy). Densitometric analysis was performed using Image J software for Windows (National Institutes of Health, Bethesda, MD, USA).

### 4.3. Immunocytochemistry and Confocal Analysis

The cells were grown on coverslips and fixed with 4% PFA in PBS, followed by permeabilization with 0.1% Triton X-100 in PBS for 5 min at room temperature, which was blocked in Bovine Serum Albumine (BSA) dissolved in 0.1% PBS Triton for 30 min. The β-III-Tubulin (Santa Cruz Biotechnology, Santa Cruz, CA, USA) and LC3 (Sigma Aldrich, Milan, Italy) primary antibodies were incubated overnight at 4 °C and visualized by means of Alexa 488 Fluor secondary antibodies (ThermoFisher Scientific, Waltham, MA, USA). After nuclear staining with DAPI (ThermoFisher Scientific, Waltham, MA, USA), the coverslips were mounted with Fluoroshield mounting medium (F6182, Merck Life Science, Milan, Italy) and examined under a confocal microscope (TCS SP8; Leica, Wetzlar, Germany). Leica Application Suite X software equipped with a 40 × 1.40–0.60 NA HCX Plan Apo oil BL objective at RT was used for image acquisition and analysis. 

### 4.4. Morphological Analysis 

The lengths of the cytoplasmic extensions were evaluated at different time intervals and for each experimental group at different fields derived from observation under the light microscope (Eclipse 7s100; Nikon Europe, Amstelveen, The Netherlands) at 20× magnification. Morphological analysis was performed with ImageJ software. The lengths of the cytoplasmic extensions are expressed in arbitrary units.

### 4.5. Proliferation and Apoptosis Assays

The GBM cells were grown on 12-well plates in the presence of the indicated stimuli or of the vehicle. Cell proliferation was assessed by counting cells in a THOMA chamber at the indicated time points after trypsinization. At least three counts for each condition were performed in each experiment. For apoptosis detection by DAPI staining of the nuclei, the cells were grown on coverslips and fixed with 4% PFA in PBS followed by DAPI staining. The fragmented and condensed nuclei (apoptotic) and the intact nuclei were counted under a confocal microscope as described above. At least 10 fields at 20× magnification were counted. FACS analysis by flow cytometry was also performed to detect the subG1 and apoptotic cells. The cells were plated in 35 mm plates in complete medium and treated with JQ1, TMZ, or their combination for 48 h. After treatment, the cells were trypsinised, washed in sample buffer, and fixed in a cold methanol/acetone (4:1) solution. Before analysis, 250 μM of propidium iodide was added for 30 min. Flow cytometry (FACS) analysis of the cell cycle was performed using an FACSCalibur (BD Biosciences, Franklin Lakes, NJ, USA).

### 4.6. Statistical Analysis

All experiments were performed at least three times. GraphPad Prism software (GraphPad, La Jolla, CA, USA) was used for the statistical analysis. All results presented in this study are expressed as means ± SDs (standard deviations). Statistical significance was determined, as indicated, by using one-way ANOVA followed by a Tukey’s post hoc test or two-way analysis of variance (ANOVA) followed by a Bonferroni’s post hoc test. For immunofluorescence analyses, Student’s *t*-tests were used. *p*-values of ≤0.05 were considered significant. 

## Data Availability

Not applicable.

## Supplementary Materials

The following supporting information can be downloaded at: https://www.mdpi.com/article/10.3390/ijms24087017/s1.

## References

[1] Louis D.N., Perry A., Reifenberger G., von Deimling A., Figarella-Branger D., Cavenee W.K., Ohgaki H., Wiestler O.D., Kleihues P., Ellison D.W. (2016). The 2016 World Health Organization Classification of Tumors of the Central Nervous System: A summary. Acta Neuropathol..

[2] Wen P.Y., Weller M., Lee E.Q., Alexander B.M., Barnholtz-Sloan J.S., Barthel F.P., Batchelor T.T., Bindra R.S., Chang S.M., Antonio Chiocca E. (2020). Glioblastoma in adults: A Society for Neuro-Oncology (SNO) and European Society of Neuro-Oncology (EANO) consensus review on current management and future directions. Neuro. Oncol..

[3] Janjua T.I., Rewatkar P., Ahmed-Cox A., Saeed I., Mansfeld F.M., Kulshreshtha R., Kumeria T., Ziegler D.S., Kavallaris M., Mazzieri R. (2021). Frontiers in the treatment of glioblastoma: Past, present and emerging. Adv. Drug Deliv. Rev..

[4] Khaddour K., Johanns T.M., Ansstas G. (2020). The landscape of novel therapeutics and challenges in glioblastoma multiforme: Contemporary state and future directions. Pharmaceuticals.

[5] Weller M., Cloughesy T., Perry J.R., Wick W. (2013). Standards of care for treatment of recurrent glioblastoma-are we there yet?. Neuro. Oncol..

[6] Witthayanuwat S., Pesee M., Supaadirek C., Supakalin N., Thamronganantasakul K., Krusun S. (2018). Survival analysis of Glioblastoma Multiforme. Asian Pacific J. Cancer Prev..

[7] Gimple R.C., Bhargava S., Dixit D., Rich J.N. (2019). Glioblastoma stem cells: Lessons from the tumor hierarchy in a lethal cancer. Genes Dev..

[8] Mattei V., Santilli F., Martellucci S., Monache S.D., Fabrizi J., Colapietro A., Angelucci A., Festuccia C. (2021). The importance of tumor stem cells in glioblastoma resistance to therapy. Int. J. Mol. Sci..

[9] Osuka S., Van Meir E.G. (2017). Overcoming therapeutic resistance in glioblastoma: The way forward. J. Clin. Investig..

[10] Galli R., Binda E., Orfanelli U., Cipelletti B., Gritti A., De Vitis S., Fiocco R., Foroni C., Dimeco F., Vescovi A. (2004). Isolation and characterization of tumorigenic, stem-like neural precursors from human glioblastoma (Cancer Research (October 2004) 64 (7011–7021). Cancer Res..

[11] Piccirillo S.G.M., Binda E., Fiocco R., Vescovi A.L., Shah K. (2009). Brain cancer stem cells. J. Mol. Med..

[12] Jin J., Grigore F., Chen C.C., Li M. (2021). Self-renewal signaling pathways and differentiation therapies of glioblastoma stem cells (Review). Int. J. Oncol..

[13] Nicholson J.G., Fine H.A. (2021). Diffuse glioma heterogeneity and its therapeutic implications. Cancer Discov..

[14] Pastori C., Daniel M., Penas C., Volmar C.H., Johnstone A.L., Brothers S.P., Graham R.M., Allen B., Sarkaria J.N., Komotar R.J. (2014). BET bromodomain proteins are required for glioblastoma cell proliferation. Epigenetics.

[15] Du Z., Song X., Yan F., Wang J., Zhao Y., Liu S. (2018). Genome-wide transcriptional analysis of BRD4-regulated genes and pathways in human glioma U251 cells. Int. J. Oncol..

[16] Tao Z., Li X., Wang H., Chen G., Feng Z., Wu Y., Yin H., Zhao G., Deng Z., Zhao C. (2020). BRD4 regulates self-renewal ability and tumorigenicity of glioma-initiating cells by enrichment in the Notch1 promoter region. Clin. Transl. Med..

[17] Delmore J.E., Issa G.C., Lemieux M.E., Rahl P.B., Shi J., Jacobs H.M., Kastritis E., Gilpatrick T., Paranal R.M., Qi J. (2011). BET bromodomain inhibition as a therapeutic strategy to target c-Myc. Cell.

[18] Bandopadhayay P., Piccioni F., O’Rourke R., Ho P., Gonzalez E.M., Buchan G., Qian K., Gionet G., Girard E., Coxon M. (2019). Neuronal differentiation and cell-cycle programs mediate response to BET-bromodomain inhibition in MYC-driven medulloblastoma. Nat. Commun..

[19] Aird F., Kandela I., Mantis C., Iorns E., Denis A., Williams S.R., Perfito N., Errington T.M. (2017). Replication study: BET bromodomain inhibition as a therapeutic strategy to target c-Myc. Elife.

[20] Jermakowicz A.M., Rybin M.J., Suter R.K., Sarkaria J.N., Zeier Z., Feng Y., Ayad N.G. (2021). The novel BET inhibitor UM-002 reduces glioblastoma cell proliferation and invasion. Sci. Rep..

[21] Lam F.C., Morton S.W., Wyckoff J., Vu Han T.L., Hwang M.K., Maffa A., Balkanska-Sinclair E., Yaffe M.B., Floyd S.R., Hammond P.T. (2018). Enhanced efficacy of combined temozolomide and bromodomain inhibitor therapy for gliomas using targeted nanoparticles. Nat. Commun..

[22] Berenguer-Daizé C., Astorgues-Xerri L., Odore E., Cayol M., Cvitkovic E., Noel K., Bekradda M., MacKenzie S., Rezai K., Lokiec F. (2016). OTX015 (MK-8628), a novel BET inhibitor, displays in vitro and in vivo antitumor effects alone and in combination with conventional therapies in glioblastoma models. Int. J. Cancer.

[23] Tancredi A., Gusyatiner O., Bady P., Buri M.C., Lomazzi R., Chiesi D., Messerer M., Hegi M.E. (2022). BET protein inhibition sensitizes glioblastoma cells to temozolomide treatment by attenuating MGMT expression. Cell Death Dis..

[24] Sakamaki J.I., Wilkinson S., Hahn M., Tasdemir N., O’Prey J., Clark W., Hedley A., Nixon C., Long J.S., New M. (2017). Bromodomain Protein BRD4 Is a Transcriptional Repressor of Autophagy and Lysosomal Function. Mol. Cell.

[25] Simpson J.E., Gammoh N. (2020). The impact of autophagy during the development and survival of glioblastoma: Role of autophagy in glioblastoma. Open Biol..

[26] Batara D.C.R., Choi M.C., Shin H.U., Kim H., Kim S.H. (2021). Friend or foe: Paradoxical roles of autophagy in gliomagenesis. Cells.

[27] Colella B., Faienza F., Di Bartolomeo S. (2019). EMT regulation by autophagy: A new perspective in glioblastoma biology. Cancers.

[28] Colella B., Colardo M., Iannone G., Contadini C., Saiz-Ladera C., Fuoco C., Barilà D., Velasco G., Segatto M., Di Bartolomeo S. (2020). Mtor inhibition leads to src-mediated egfr internalisation and degradation in glioma cells. Cancers.

[29] Colardo M., Segatto M., Di Bartolomeo S. (2021). Targeting rtk-pi3k-mtor axis in gliomas: An update. Int. J. Mol. Sci..

[30] Ryskalin L., Gaglione A., Limanaqi F., Biagioni F., Familiari P., Frati A., Esposito V., Fornai F. (2019). The autophagy status of cancer stem cells in gliobastoma multiforme: From cancer promotion to therapeutic strategies. Int. J. Mol. Sci..

[31] Ferrucci M., Biagioni F., Lenzi P., Gambardella S., Ferese R., Calierno M.T., Falleni A., Grimaldi A., Frati A., Esposito V. (2017). Rapamycin promotes differentiation increasing βIII-tubulin, NeuN, and NeuroD while suppressing nestin expression in glioblastoma cells. Oncotarget.

[32] Brunel A., Hombourger S., Barthout E., Battu S., Kögel D., Antonietti P., Deluche E., Saada S., Durand S., Lalloué F. (2021). Autophagy inhibition reinforces stemness together with exit from dormancy of polydisperse glioblastoma stem cells. Aging.

[33] Catalano M., D’Alessandro G., Lepore F., Corazzari M., Caldarola S., Valacca C., Faienza F., Esposito V., Limatola C., Cecconi F. (2015). Autophagy induction impairs migration and invasion by reversing EMT in glioblastoma cells. Mol. Oncol..

[34] Filippakopoulos P., Qi J., Picaud S., Shen Y., Smith W.B., Fedorov O., Morse E.M., Keates T., Hickman T.T., Felletar I. (2010). Selective inhibition of BET bromodomains. Nature.

[35] Taniguchi Y. (2016). The bromodomain and extra-terminal domain (BET) family: Functional anatomy of BET paralogous proteins. Int. J. Mol. Sci..

[36] Filippakopoulos P., Picaud S., Mangos M., Keates T., Lambert J.P., Barsyte-Lovejoy D., Felletar I., Volkmer R., Müller S., Pawson T. (2012). Histone recognition and large-scale structural analysis of the human bromodomain family. Cell.

[37] Cheung K.L., Kim C., Zhou M.M. (2021). The Functions of BET Proteins in Gene Transcription of Biology and Diseases. Front. Mol. Biosci..

[38] Li J., Yang B., Zhou Q., Wu Y., Shang D., Guo Y., Song Z. (2018). Autophagy promotes hepatocellular carcinoma cell invasion through activation of epithelial—Mesenchymal transition. Carcinogenesis.

[39] Tian T., Guo T., Zhen W., Zou J., Li F. (2020). BET degrader inhibits tumor progression and stem-like cell growth via Wnt/β-catenin signaling repression in glioma cells. Cell Death Dis..

[40] Li J., Ma J., Meng G., Lin H., Wu S., Wang J., Luo J., Xu X., Tough D., Lindon M. (2016). BET bromodomain inhibition promotes neurogenesis while inhibiting gliogenesis in neural progenitor cells. Stem Cell Res..

[41] Nakano I. (2015). Stem cell signature in glioblastoma: Therapeutic development for a moving target. J. Neurosurg..

[42] Li F., Yang C., Zhang H.B., Ma J., Jia J., Tang X., Zeng J., Chong T., Wang X., He D. (2019). BET inhibitor JQ1 suppresses cell proliferation via inducing autophagy and activating LKB1/AMPK in bladder cancer cells. Cancer Med..

[43] Di Bartolomeo S., Nazio F., Cecconi F. (2010). The Role of Autophagy During Development in Higher Eukaryotes. Traffic.

[44] Rodolfo C., Di Bartolomeo S., Cecconi F. (2016). Autophagy in stem and progenitor cells. Cell. Mol. Life Sci..

[45] Adelipour M., Saleth L.R., Ghavami S., Alagarsamy K.N., Dhingra S., Allameh A. (2022). The role of autophagy in the metabolism and differentiation of stem cells. Biochim. Biophys. Acta Mol. Basis Dis..

[46] Klionsky D.J., Abdelmohsen K., Abe A., Abedin M.J., Abeliovich H., Arozena A.A., Adachi H., Adams C.M., Adams P.D., Adeli K. (2016). Guidelines for the use and interpretation of assays for monitoring autophagy (3rd edition). Autophagy.

