# Supplementary material for: Bromodomain and Extraterminal Domain (BET) Protein Inhibition Hinders Glioblastoma Progression by Inducing Autophagy-Dependent Differentiation
Source: Int J Mol Sci. 2023 Apr 10;24(8):7017. doi: 10.3390/ijms24087017 (PMC10138987; doi:10.3390/ijms24087017)
Supplement: Supplementary file 1 [file ijms-24-07017-s001.zip › ijms-2272465-supplementary-update.pdf]

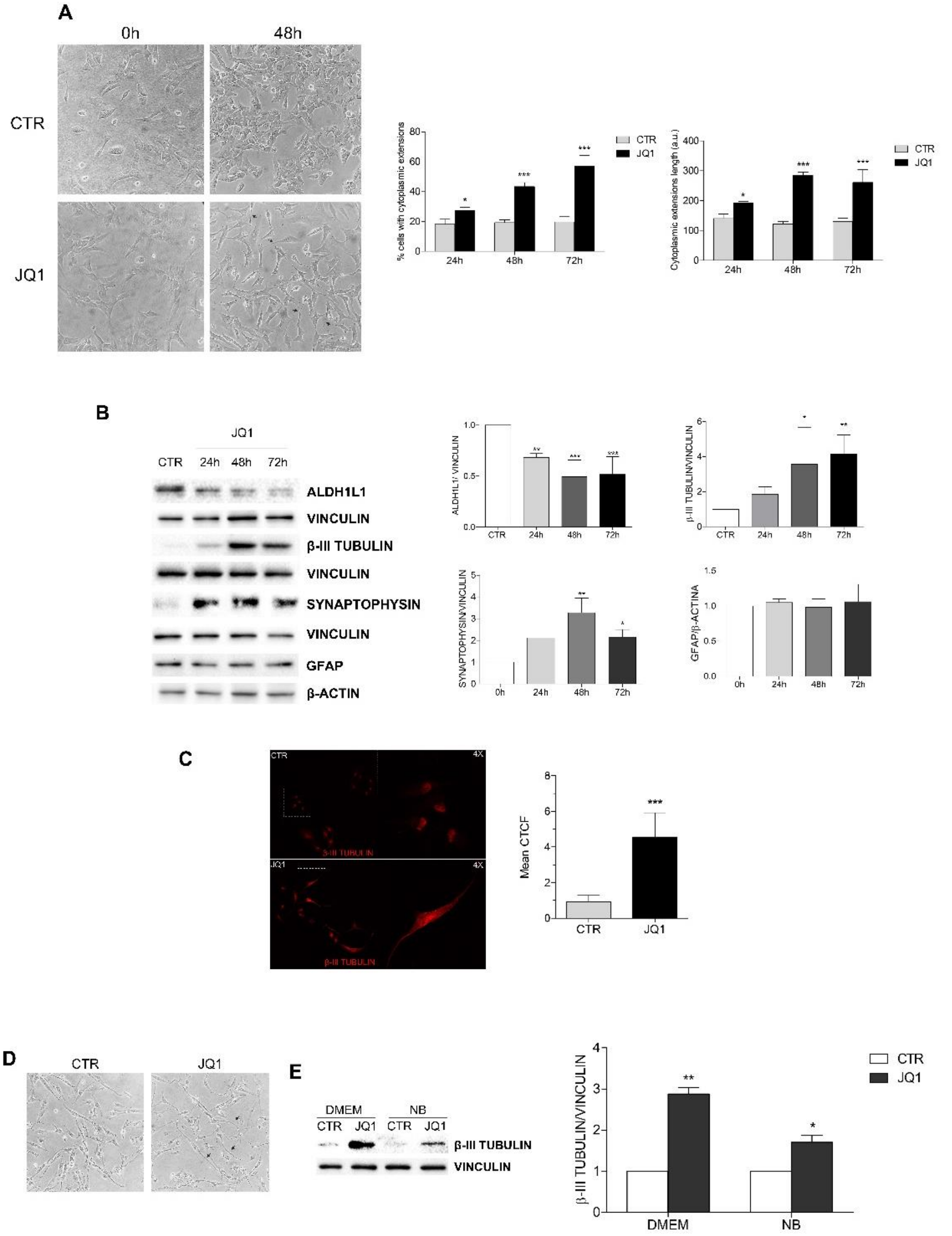

Supplementary Figure S1. BET inhibition induces neuronal-like differentiation in GH2 primary cells. (A) GH2 cells were cultured in complete DMEM (CTR) or in presence of 0,5 M JQ1 and phase contrast pictures, at 20x magnification, were taken at 24, 48 and 72 hours. Representative images of cells upon 48h of JQ1 stimulation are shown. Arrow heads indicate cytoplasmic extensions. Scale bar: 100 μm. The percentage of cells with cytoplasmic extensions and the extension lenght were calculated by Image J analysis and shown in the graphs. At least 10 fields per each condition were counted. ** P < .01, *** P ≤ .001 Two-way ANOVA. **(B)** Western botting analysis was performed on protein extracts from GH2 cells cultured as in (A). Specific antibodies for ALDH1, 3-TUBULIN, SYNAPTOPHISIN and GFAP were used and representative images are shown. GAPDH, VINCULIN and -TUBULIN were used as loading controls. The corresponding densitometric analyses are shown in the graphs. Statistical significance: * P < .05 ** P < .01 *** P ≤ .001 One-way ANOVA. **(C)** Immunocytochemistry and confocal analysis for 3-TUBULIN expression (red) was performed in GH2 cells, upon 48h of 0,5 M JQ1 treatment. Scale bar, 30 μM. A 4X magnification is shown in the right panel. Analysis of the mean Corrected Total Cell Fluorescence (CTCF) was performed by Image J software on 10 fields per each condition and the corresponding graph is shown. *** P ≤ .001 One-way ANOVA. **(D)** GH2 cells were cultured, in monolayer, in Neurobasal/B27 medium

for 8 hours before stimulation with 0,5 M JQ1. After 48 hours, phase contrast pictures, at 20x magnification were taken. Arrow heads in the pictures indicate cytoplasmic extensions. Scale bar: 100 µm. (E) GH2 cells grown in DMEM or in Neurobasal medium (NB) were treated with 0.5 µM JQ1 for 48 h. The corresponding protein extracts were subjected to western blotting analysis for βIII-TUBULIN and Vinculin was used as loading control. Densitometric analysis of the blot is shown in the graph. * P < 0.05, ** P < 0.01, one-way ANOVA.

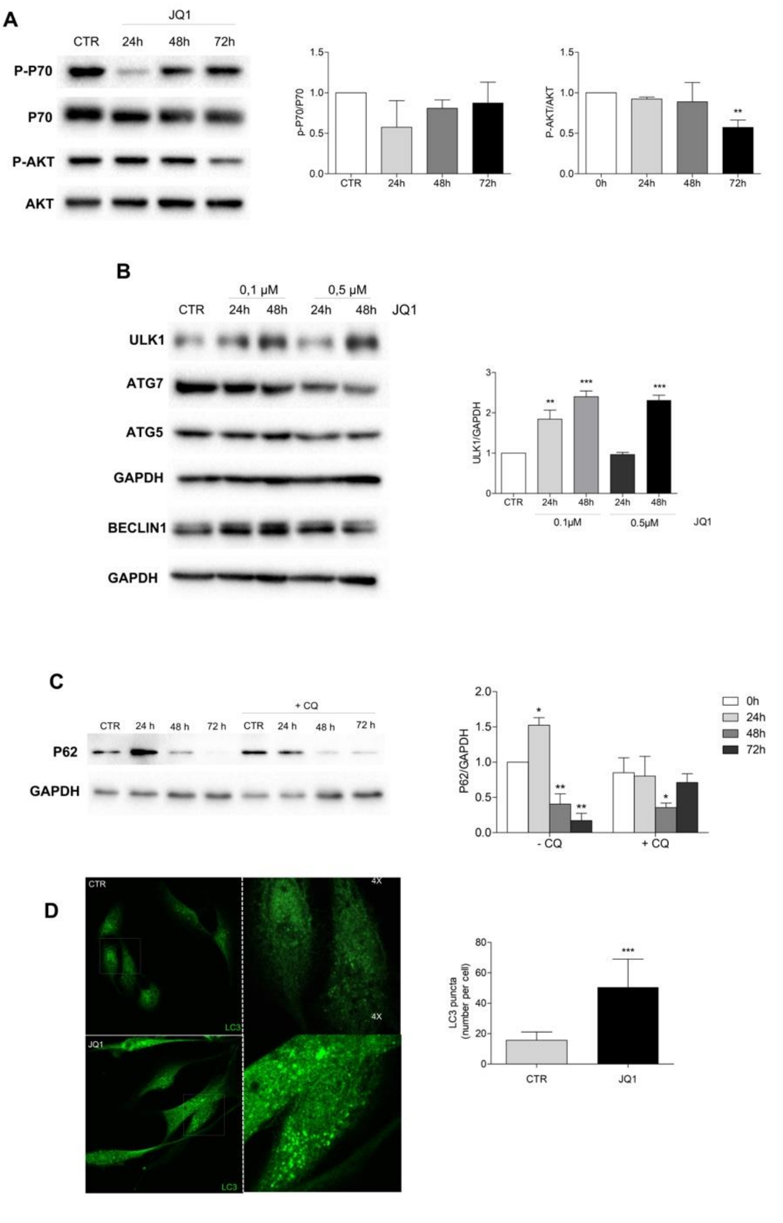

Supplementary Figure S2. Autophagy is induced in JQ1-stimulated GH2 cells. (A). Western blotting analysis of the autophagy regulators ULK1, ATG7, ATG5 and BECLIN1 in protein ectracts from U87 cells stimulated with 0,1 or 0,5 mM JQ1 for 24 and 48 hours. Representative images of three different experiments are shown. GAPDH was used as loading control. The corresponding densitometric analyses are shown in the graphs. Statistical significance: ** P < .01 *** P ≤ .001 Two-way ANOVA **(B)** Autophagy flux was analyzed in cells stimulated as in (A) in presence or absence of 20 M Choloquine (CQ). The expression of p62 is shown. VINCULIN was used as loading control. The corresponding densitometric analyses are shown. Statistical significance: * P < .05 ** P < .01 Two-way ANOVA. **(C)** U87 treated with or 0,5 mM JQ1 for 48 hours or untreated (CTR) were subjected to immunocytochemistry and confocal analysis for LC3 (green) and

LAMP2 (red). Representative images of at least 10 fields, showing the merge of the two signals, are shown. Scale bar, 30 μM. A 4X magnification is shown in the right panel. LC3 puncta per cell were counted in 30 cells for each condition and the correponding graph is shown. Statistical significance: *** $P \leq .001$ Student t-test. Colocalisation was assessed by calculating the Pearson's correlation coefficient r and it is shown in the right graph. Statistical significance: * $P < .05$ ** $P < .01$ Student t-test. (**D**) GH2 cells treated with 0.5 M of JQ1 for 48 h and untreated (CTR) cells were subjected to immunocytochemistry and confocal analysis for LC3 (green). Representative images of at least 10 fields showing the merged signals are shown. Scale bar: 30 μM. A 4× magnification is shown in the right panel. The LC3 puncta per cell were counted in 30 cells for each condition, and the corresponding graph is shown. Statistical significance: *** $P \leq 0.001$, Student's t-test.
